# The PPARγ pathway determines electrophysiological remodelling and arrhythmia risks in DSC2 arrhythmogenic cardiomyopathy

**DOI:** 10.1002/ctm2.748

**Published:** 2022-03-16

**Authors:** Jean‐Baptiste Reisqs, Adrien Moreau, Azzouz Charrabi, Yvonne Sleiman, Albano C. Meli, Gilles Millat, Veronique Briand, Philippe Beauverger, Sylvain Richard, Philippe Chevalier

**Affiliations:** ^1^ Neuromyogene Institute Claude Bernard University, Lyon 1, Villeurbanne France; ^2^ Cardiovascular and Metabolism Research Sanofi R&D, Chilly Mazarin France; ^3^ Université de Montpellier, INSERM, CNRS, PhyMedExp, Montpellier France; ^4^ Hospices Civils de Lyon, Lyon Service de Rythmologie France; ^5^ Laboratoire de Cardiogénétique Moléculaire Centre de Biologie et Pathologie Est Bron France

Dear Editor,

Arrhythmogenic cardiomyopathy (ACM) is a rare, life‐threatening genetic disease frequently associated with mutations in desmosomal genes.^1^ Histopathological hallmark includes fibrofatty replacement of myocardial tissue, potentially consisting of cardiomyocytes transdifferentiation into adipocytes.^2^ The ACM involves electromechanical disorders and risks of developing arrhythmias and sudden cardiac death. We recently evidenced early electrical modifications of human‐induced pluripotent stem cells‐derived cardiomyocytes (hiPSC‐CM) obtained from an ACM patient with a missense mutation (c.394C>T) in the *DSC2* gene encoding desmocollin 2 (DSC2‐hiPSC‐CMs). These modifications are risk factors for triggering arrhythmias independently of fibrofatty replacement of myocardial tissue.^3^ We now show that PPARγ, a master regulator of the cardiomyocytes transdifferentiation into adipocytes, is critical early in the pro‐arrhythmogenic pathogenesis in ACM‐DSC2‐hiPSC‐CMs.

Reverse transcription‐quantitative polymerase chain reaction (RT‐qPCR) analysis of heart samples revealed a higher PPARγ gene expression level in the ACM‐heart bearing the *DSC2* mutation than in the control heart (Figure S1). We derived hiPSCs from the same ACM‐DSC2 patient, differentiated them into hiPSC‐CMs, and cultured them for 60 days as described.^3^ We found a similar higher expression of PPARγ and of two pro‐adipogenic target genes, perilipin and adiponectin, in DSC2‐hiPSC‐CMs versus control‐hiPSC‐CMs (Figure 1A–C). Despite lower expression, the pro‐cardiac myosin light chain 2 ventricular (MLC2v) validated the cardiomyocyte phenotype of DSC2‐hiPSC‐CMs (Figure 1D). To confirm the involvement of PPARγ in the genes switch, we challenged DSC2‐hiPSC‐CMs with T0070907 (T007), which functions as a transcriptionally corepressor‐selective PPARγ inverse agonist.^4^ Incubation with T007 (1 μM), for 40 days (D20–D60) during the maturation phase, corrected the expression levels of PPARγ, perilipin, adiponectin and MLC2v (Figure 1A–D). These results corroborated the PPARγ‐dependent pro‐adipogenic switch in the DSC2‐hiPSC‐CMs, linking with previous studies.^2^, ^5^, ^6^

**FIGURE 1:**
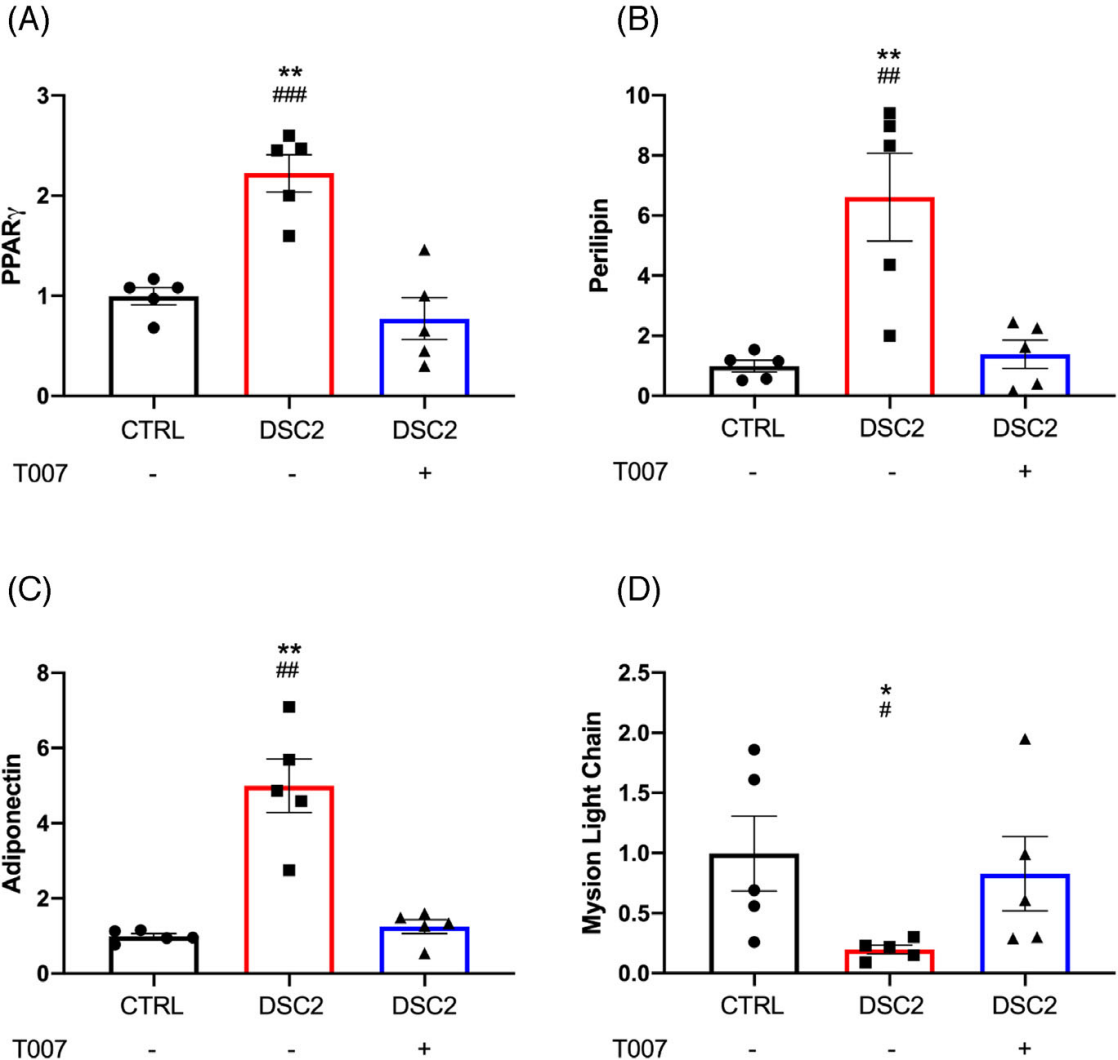
T0070907 modulates PPARγ pathway and prevents the cardiomyocyte phenotype in DSC2 patient‐specific human‐induced pluripotent stem cells‐derived cardiomyocytes (hiPSC‐CM). Fold change compared to control for (A) PPARγ, (B) perilipin, (C) adiponectin and (D) myosin light chain (MLC) expression level, measured using reverse transcription‐quantitative polymerase chain reaction (RT‐qPCR). Error bars represent the standard error of the mean (SEM), *N* = 5/group. ^*^Control versus DSC2, ^#^DSC2 versus T007; ^*,#^ *p*< .05, ^**,##^ *p*< .01, ^###^ *p* < .001 (Tuckey multiple comparisons test)

We next investigated the effects of T007 on the excitation–contraction coupling using a phase‐contrast video‐based analysis of the hiPSC‐CM monolayer's contractile function. We defined several contractile parameters: the beat rate, contraction time, relaxation duration, the resting time between two contractions, asynchronous rate and differentiating factor (Figure 2A–F). For each analysed video, the asynchronous time defines the time‐fraction of the area spent in asynchrony. The differentiating factor reflects the linear combination of determinant contractile properties ensuring the best discrimination between the control‐hiPSC‐CMs and the DSC2‐hiPSC‐CMs.

**FIGURE 2:**
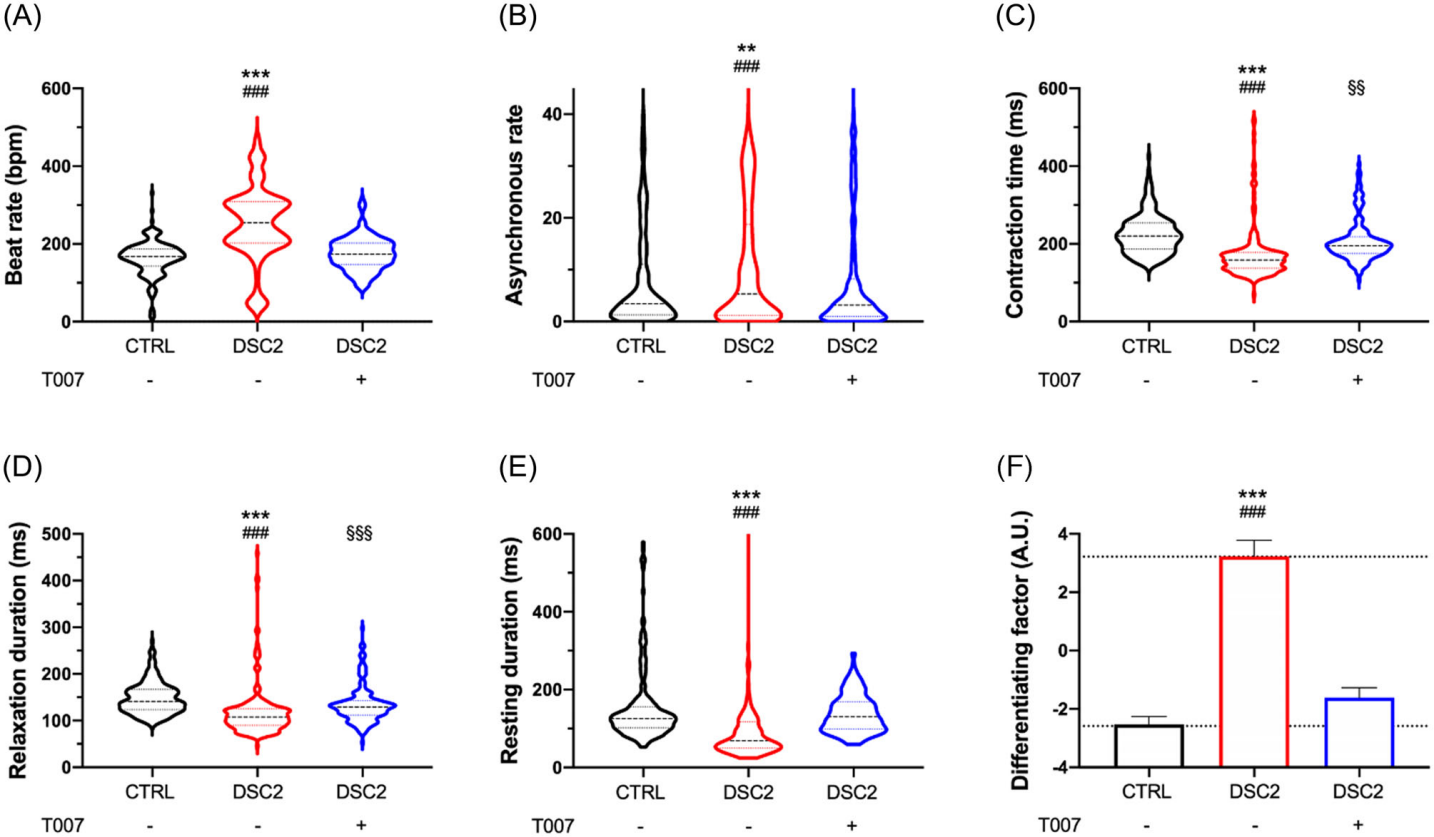
T0070907 prevents contractile disturbances in DSC2 patient‐specific human‐induced pluripotent stem cells‐derived cardiomyocytes (hiPSC‐CMs). The contractile activity was studied by video analysis in monolayer condition (control, *N* = 249 (black); patient, *N* = 212 (red); T0070907, *N* = 197 (blue)): beat rate (A), asynchronous rate (B), contraction time (C), relaxation duration (D), resting duration (E) and differentiating factor (F). Results are represented by violin plot with the median, first and third quartile. ^*^Control versus DSC2, ^#^DSC2 versus T007, ^§^control versus T007; ^**,§§^ *p* < .01, ^***,###,§§§^ *p* < .001 (Kruskal–Wallis test)

The mutation increased the beat and asynchronous rates (Figure 2A,B) and reduced the contraction time, relaxation and resting duration in DSC2‐hiPSC‐CMs versus control‐hiPSC‐CMs (Figure 2C–E). Incubation with T007 prevented these modifications. The differentiating factor was similar to that of the control‐hiPSC‐CMs (Figure 2F), showing that PPARγ pathway inhibition counteracts the pathogenesis process. Interestingly, the PPARγ agonist (GW1929) did not mimic the effect of the mutation on the differentiating factor and action potential (AP) duration in control‐hiPSC‐CMs (Figure S2A) in line with the negligible effect of PPARγ agonists on basal transcription.^4^ A significant PPARγ expression level may be required for ligand‐mediated effects.

In cardiomyocytes, the AP initiates the contraction. We studied the effect of T007 on the electrophysiological properties of DSC2‐hiPSC‐CMs using the patch‐clamp technique. Compared to control‐hiPSC‐CMs, the DSC2‐hiPSC‐CMs exhibited a twofold shortening of the AP duration at 1.0 Hz and lost action potential duration (APD) modulation according to pacing rate (Figure 3A,B). The voltage‐gated Na^+^ current (*I*_Na_) induces AP fast depolarisation, playing a master role in cardiac excitability. The DSC2‐hiPSC‐CMs exhibited a twofold decrease in *I*_Na_ density and a rightward shift in its voltage‐dependent activation and steady‐state inactivation (Figures 3C,D and S3). Consistently, the expression level of the *SCN5A* gene coding for the cardiac Na_v_ channels (*I*_Na_) was decreased (Figure S4). Voltage‐gated K^+^ currents play a crucial role in AP repolarisation. Compared to control‐hiPSC‐CMs, DSC2‐hiPSC‐CMs exhibited a 3.5‐fold increase in K^+^ current density, accounting for the AP shortening (Figure 3E,F). RT‐qPCR analysis revealed an increase of +227% in the *KCNH2* gene coding for *I*_Kr_ and +220% in the *KCNQ1* gene coding for *I*_Ks_ in DSC2‐hiPSC‐CMs compared to control‐hiPSC‐CMs (Figure S4). Exposure of DSC2‐hiPSC‐CMs to T007 prevented the differences with control‐hiPSC‐CMs (Figures 3A–F and S3–S5).

**FIGURE 3:**
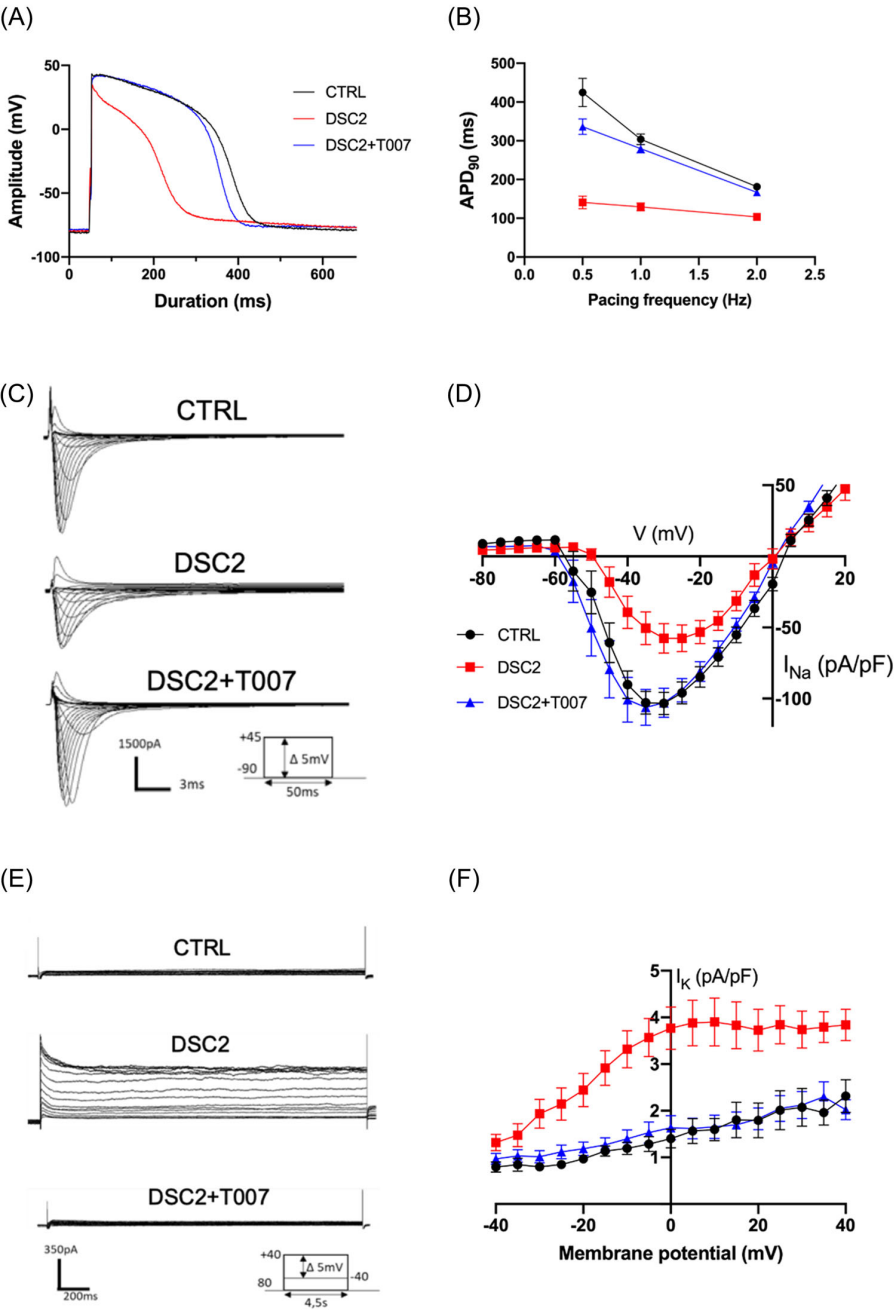
Electrical activity of control and patient‐specific human‐induced pluripotent stem cells‐derived cardiomyocytes (hiPSC‐CMs) with or without T0070907. (A) Action potentials (APs) of hiPSC‐CM from control (black), DSC2 patient in absence of T0070907 (red) and after 40 days of incubation with 1 μM of T0070907 (blue). (B) AP duration at 90% of repolarisation (APD_90_) as a function of pacing rate (control, *N* = 42; DSC2, *N* = 40; DSC2 + T0070907, *N* = 40). (C) Whole‐cell Na^+^ current traces of control (top), patient‐specific hiPSC‐CMs (middle) and after incubation of T007 (bottom). (D) Averaged current density–voltage (*I*–*V*) relationship of *I*_Na_ (control, *N* = 23; DSC2, *N* = 21; DSC2 + T0070907, *N* = 23). (E) Whole‐cell *I*_K_ currents raw traces of control (top), patient‐specific (middle) and after incubation of T0070907 (bottom) hiPSC‐CMs. (F) *I*–*V* curves of *I*_K_ associated currents (control, *N* = 15; DSC2, *N* = 18; DSC2 + T007, *N* = 14). Error bars represent the standard error of the mean (SEM). All differences are statistically different, *p* < .001, DSC2 versus control and versus DSC2 + T0070907 (Tuckey multiple comparison test)

We evaluated the Ca^2+^ transients of spontaneously beating hiPSC‐CMs using the fluorescent non‐ratiometric Fluo‐4 Ca^2+^‐sensitive dye. The DSC2‐hiPSC‐CMs (vs. control‐hiPSC‐CMs) exhibited increased Ca^2+^ transient frequency but decreased decay time and global Ca^2+^ mobilised area under the curve (AUC) during the Ca^2+^ transient (Figure 4A–C) in coherence with impaired contractility. They also exhibited an increase in the number and frequency of pro‐arrhythmogenic abnormal spontaneous diastolic microscopic Ca^2+^ events (Ca^2+^ sparks), reflecting Ca^2+^ leak from the sarcoplasmic reticulum through the ryanodine receptor. Treatment of the DSC2‐hiPSC‐CMs with T007 attenuated all these effects (Figure 4D,E).

**FIGURE 4:**
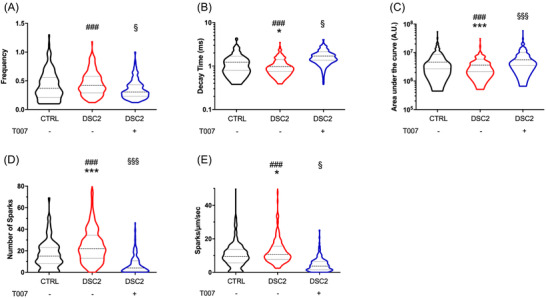
Spontaneous Ca^2+^ handlings of control, patient‐specific human‐induced pluripotent stem cells‐derived cardiomyocytes (hiPSC‐CMs) and incubated with T0070907. The Ca^2+^ transient activity (control, *N* = 207 (black); patient, *N* = 222 (red); T0070907, *N* = 197 (blue)) were studied. Spontaneous frequency (A), decay time (B) and area under the curve (AUC) (C). The Ca^2+^ sparks were studied by measuring the number of sparks in each cell (D) and the frequency of sparks (E). The violin plot represented the median, first and third quartile, and a Kruskal–Wallis test was performed. ^*^Control versus DSC2, ^#^DSC2 versus T007, ^§^control versus T007; ^*,§^ *p* < .05, ^***,###,§§§^ *p* < .001 (Tuckey multiple comparisons test were performed)

Late T007 incubation (D55–D60) could not reverse the variant‐specific modifications of the AP in DSC2‐hiPSC‐CMs (Figure S5). In addition, when we cultured DSC2‐hiPSC‐CMs in the presence of T007 between D20 and D60 and then removed the drug at D100, the cells exhibited the typical disease phenotype (Figure S5). Therefore, early and continuous exposure of DSC2‐hiPSC‐CMs to T007 is required to maintain a wild‐type‐like cellular electrophysiological signature (Figure S6).

In conclusion, the repressing effect of T007 on PPARγ transcriptional activity maintained regular electrical activity and Ca^2+^ handling in hiPSC‐CMs bearing the *DSC2* (c.394C>T) mutation. T007 also lowered early pro‐arrhythmogenic events intrinsic to cardiomyocytes, likely to contribute to rhythm disturbance independently of fibrofatty replacement of myocardial tissue in ACM patients (Figure S7). The risk of malignant ventricular tachyarrhythmias relies both on (i) shortened AP repolarisation, responsible for short QT (and JTc) intervals in a cohort of patients,^3^ caused by high *KCNH2* (*I*_Kr_) and *KCNQ1* (*I*_Ks_) expressions and (ii) and abnormal occurrence of Ca^2+^ sparks known to promote arrhythmias independently of any change in the AP and QT interval.^7^ Combining a short AP (QT) with disturbed Ca^2+^ is likely to increase the pro‐arrhythmogenic risk. Our results align with different studies in transgenic mice linking PPARγ activation, cardiomyocyte lipid accumulation, dilated cardiomyopathy, changes in electrophysiological profile and intracellular Ca^2+^ handling, and arrhythmogenic risks, although differences between mice and humans may limit some interpretations.^6^, ^8^, ^9^, ^10^ Overall, our study provides new comprehensive insights directly in human cardiomyocytes regarding the role of PPARγ in genetic ACM. Repression of PPARγ transcription may offer a unique opportunity for ACM treatment.

## FUNDING INFORMATION

A CIFRE grant from Sanofi R&D supported this work to Jean‐Baptiste Reisqs. Fond Marion Elisabeth Brancher supported the post‐doctoral fellowship of Adrien Moreau.

## CONFLICT OF INTERESTS

Sanofi, a global biopharmaceutical company focused on human health, employs Jean‐Baptiste Reisqs, Veronique Briand and Philippe Beauverger. All other authors have nothing else to disclose. All authors read and approved the final version of the manuscript and ensure it was the case.

## Supporting information

Supporting Information
Figure S1: PPARγ expression in patient DSC2 heart sample. Relative level of PPARγ in human heart sample from control and an arrhythmogenic cardiomyopathy (ACM) patient with a missense mutation (c.394C>T) in the *DSC2* gene. Data obtained from transcriptome analysis (Affymetrix, HG‐U133_plus2 chips)
Figure S2: Evaluation of PPARγ agonist in WT human‐induced pluripotent stem cells‐derived cardiomyocytes (hiPSC‐CM). (A) Differentiating factor of contractile property and (B) action potential (AP) duration at 90% of repolarisation (APD_90_) in WT hiPSC‐CM with or without 40 days of 5 μM GW1929. ^*^Control versus DSC2, ^§^DSC2 versus GW1929; ^§§^ *p* < .01, ^***,§§§^ *p* < .001 (*t*‐test comparison)
Figure S3: Evaluation of Na_v_ biophysical parameters. (A) Voltage‐dependence of steady‐state activation and inactivation of Na_v_ channels
Figure S4: Evaluation of sodium and potassium channel expression. (A) The expression level of voltage‐gated Na^+^ channel alpha subunit (*N* = 4/group). Expression level study by reverse transcription‐quantitative polymerase chain reaction (RT‐qPCR) of potassium voltage‐gated channel subfamily H2 (B) and subfamily Q1 (C) (*N* = 4/group). Error bars represent the standard error of the mean (SEM). ^*^Control versus DSC2, ^#^DSC2 versus T007; ^*,#^ *p* < .05, ^**,##^ *p*< .01, ^***^ *p* < .001 (Tuckey multiple comparisons test)
Figure S5: Electrical activity of control and patient‐specific human‐induced pluripotent stem cells‐derived cardiomyocytes (hiPSC‐CMs) with or without PPARγ for 5 days. (A) raw trace illustrating the AP of control (black), patient‐specific hiPSC‐CM (red) and after incubation of T0070907 (grey), (B) Action potential (AP) duration at 90% of repolarisation (APD_90_), (C) adaptation of APD at 0.5, 1 and 2 Hz. Error bars represent the standard error of the mean (SEM). ^*^Control versus DSC2, ^$^control versus T007; ^*^ *p* < .05, ^***,$$$^ *p* < .001 (Tuckey multiple comparisons test)
Figure S6: Inhibition of the PPARγ pathway is reversible. (A) Raw trace illustrating the action potential (AP) of control (black), patient‐specific human‐induced pluripotent stem cells‐derived cardiomyocytes (hiPSC‐CMs) (red) and after temporary incubation with 1 μM T0070907 for 40 days (D20–D60) and then removal of the drug for the next 40 days (D60–D100) (blue). (B) AP duration at 90% of repolarisation (APD_90_). (C) Adaptation of APD_90_ at different pacing (0.5, 1.0 and 2.0 Hz). (D) Comparison of APD_90_ in control, DSC2 and DSC2 + T007 between D60 (blank) and D100 (black square). Error bars represent the mean standard error (SEM) (control, *N* = 32; DSC2, *N* = 34; DSC2 + T007, *N* = 33). ^*^Control versus DSC2, ^§^control versus T007, ^+^DSC2 + T007 D60 versus DSC2 + T007 D100 ; ^***,§§§,+++^ *p* < .001 (Tuckey multiple comparisons test)
Figure S7: A graphical picture summarising the PPARγ mechanism in arrhythmogenic cardiomyopathy (ACM) patient. The DSC2 mutation contributes to destabilising the desmosomal complex in ACM patients. PPARγ pathway contributes to adipogenic transformation and increases the risk of ventricular arrhythmias. Defects in excitation–contraction coupling were associated with cardiomyocytes transdifferentiation into adipocytes in immature human‐induced pluripotent stem cells‐derived cardiomyocytes (hiPSC‐CMs) from patients with ACM (red panel). The PPARγ inhibitor T0070907 (T007) prevented the molecular genetic expression switch between the cardiac and the pro‐adipogenic gene expression profiles. T007 maintained regular electrical activity and calcium handling in hiPSC‐CMs bearing the *DSC2* (c.394C>T) mutation (green panel)

## References

[1] Awad MM , Calkins H , Judge DP . Mechanisms of disease: molecular genetics of arrhythmogenic right ventricular dysplasia/cardiomyopathy. Nat Clin Pract Cardiovasc Med. 2008;5:258‐267. 10.1038/ncpcardio1182 18382419 PMC2822988

[2] Ma D , Wei H , Lu J , et al. Generation of patient‐specific induced pluripotent stem cell‐derived cardiomyocytes as a cellular model of arrhythmogenic right ventricular cardiomyopathy. Eur Heart J. 2013;34:1122‐1133. 10.1093/eurheartj/ehs226 22798562

[3] Moreau A , Reisqs J‐B , Delanoe‐Ayari H , et al. Deciphering DSC2 arrhythmogenic cardiomyopathy electrical instability: from ion channels to ECG and tailored drug therapy. Clin Transl Med. 2021;11:e319. 10.1002/ctm2.319 33784018 PMC7908047

[4] Brust R , Shang J , Fuhrmann J , et al. A structural mechanism for directing corepressor‐selective inverse agonism of PPARγ. Nat Commun. 2018;9:4687. 10.1038/s41467-018-07133-w 30409975 PMC6224492

[5] Garcia‐Gras E , Lombardi R , Giocondo MJ , et al. Suppression of canonical Wnt/beta‐catenin signaling by nuclear plakoglobin recapitulates phenotype of arrhythmogenic right ventricular cardiomyopathy. J Clin Invest. 2006;116:2012‐2021. 10.1172/JCI27751 16823493 PMC1483165

[6] Kim C , Wong J , Wen J , et al. Studying arrhythmogenic right ventricular dysplasia with patient‐specific iPSCs. Nature. 2013;494:105‐110. 10.1038/nature11799 23354045 PMC3753229

[7] Thireau J , Pasquié J‐L , Martel E , et al. New drugs vs. old concepts: a fresh look at antiarrhythmics. Pharmacol Ther. 2011;132:125‐145. 10.1016/j.pharmthera.2011.03.003 21420430

[8] Son N‐H , Park T‐S , Yamashita H , et al. Cardiomyocyte expression of PPARgamma leads to cardiac dysfunction in mice. J Clin Invest. 2007;117:2791‐2801. 10.1172/JCI30335 17823655 PMC1964508

[9] Morrow JP , Katchman A , Son N‐H , et al. Mice with cardiac overexpression of peroxisome proliferator‐activated receptor γ have impaired repolarization and spontaneous fatal ventricular arrhythmias. Circulation. 2011;124:2812‐2821. 10.1161/CIRCULATIONAHA.111.056309 22124376 PMC3258098

[10] Xie Y , Gu Z‐J , Wu M‐X , et al. Disruption of calcium homeostasis by cardiac‐specific over‐expression of PPAR‐γ in mice: a role in ventricular arrhythmia. Life Sci. 2016;167:12‐21. 10.1016/j.lfs.2016.10.014 27746188

