# Supplementary material for: The PPARγ pathway determines electrophysiological remodelling and arrhythmia risks in DSC2 arrhythmogenic cardiomyopathy
Source: Clin Transl Med. 2022 Mar 16;12(3):e748. doi: 10.1002/ctm2.748 (PMC8926899; doi:10.1002/ctm2.748)

**SUPPLEMENTAL DATA**

**SUPPLEMENTAL figure 1: PPARγ expression in patient DSC2 heart sample.** Relative level of PPARγ in human heart sample from control and an ACM patient with a missense mutation (c.394C>T) in the *DSC2* gene. Data obtained from transcriptome analysis (Affymetrix, HG-U133_plus2 chips).

**SUPPLEMENTAL figure 2: Evaluation of PPARγ agonist in WT hiPSC-CM.** (A) Differentiating factor of contractile property and (B) AP duration at 90% of repolarization (APD_90_) in WT hiPSC-CM with or without 40 days of 5 μM GW1929. *: Control *vs.* DSC2, §: DSC2 *vs.* GW1929. ***P<0.001 (t-test comparison).

**SUPPLEMENTAL figure 3: Evaluation of Na_v_ biophysical parameters.** (A) Voltage-dependence of steady-state activation and inactivation of Na_v_ channels.

**SUPPLEMENTAL figure 4: Evaluation of sodium and potassium channel expression.** (A) The expression level of voltage-gated Na^+^ channel alpha subunit (N=4/group). Expression level study by RT-qPCR of potassium voltage-gated channel subfamily H2 (B) and subfamily Q1 (C) (N=4/group). Error bars represent the standard error of the mean (SEM). *: Control *vs* DSC2, #: DSC2 *vs* T007, §: Control *vs* T007. ***P<0.001 (Tuckey multiple comparisons test)

**Supplemental figure 5: Electrical activity of control and patient-specific hiPSC-CM with or without PPARγ for five days**. (A) AP duration at 90% of repolarization APD90, (D) Raw trace illustrating the action potential of control (black), patient-specific hiPSC-CM (Red) and after incubation of T0070907 (grey), (E) Adaptation of APD at 0.5, 1, and 2Hz, (F) maximal upstroke velocity. Error bars represent the standard error of the mean (SEM). *: Control *vs.* DSC2, #: DSC2 *vs.* T007and $: Control *vs.* T007. ***P<0.001 (Tuckey multiple comparisons test)

**Supplemental figure 6: Inhibition of the PPARγ pathway is reversible.** (A) Raw trace illustrating the AP of control (black), patient-specific hiPSC-CMs (Red), and after temporary incubation with 1 μM T0070907 for 40 days (D20-D60) and then removal of the drug for the next 40 days (D60-D100) (blue). (B) AP duration at 90% of repolarization (APD_90_). (C) Adaptation of APD_90_ at different pacing (0.5, 1.0 and 2.0 Hz). (D) Comparaison of APD_90_ in control, DSC2, and DSC2+T007 between D60 (blank) and D100 (black square). Error bars represent the mean standard error (SEM), control n=32, DSC2 n=34 and DSC2+T007 n=33. *: Control *vs.* DSC2, # : DSC2 *vs.* T007, § : Control *vs.* T007. **+** : DSC2+T007 D60 *vs.* DSC2+T007 D100 ***P<0.001 (Tuckey multiple comparisons test).

**Supplemental figure 7: A graphical summarizing the PPARγ mechanism in ACM patient.** The DSC2 mutation contributes to destabilizing the desmosomal complex in ACM patients. PPARγ pathway contributes to adipogenic transformation and increases the risk of ventricular arrhythmias. Defects in excitation-contraction coupling were associated with cardiomyocytes transdifferentiation into adipocytes in immature hiPSC-CM from patients with Arrhythmogenic Cardiomyopathy (ACM) (Red panel). The PPARγ inhibitor T0070907 (T007) prevented the molecular genetic expression switch between the cardiac and the pro-adipogenic gene expression profiles. T007 maintained regular electrical activity and calcium handling in hiPSC-CMs bearing the *DSC2 (*c.394C>T) mutation. (Green panel).

**Supplemental data 1**


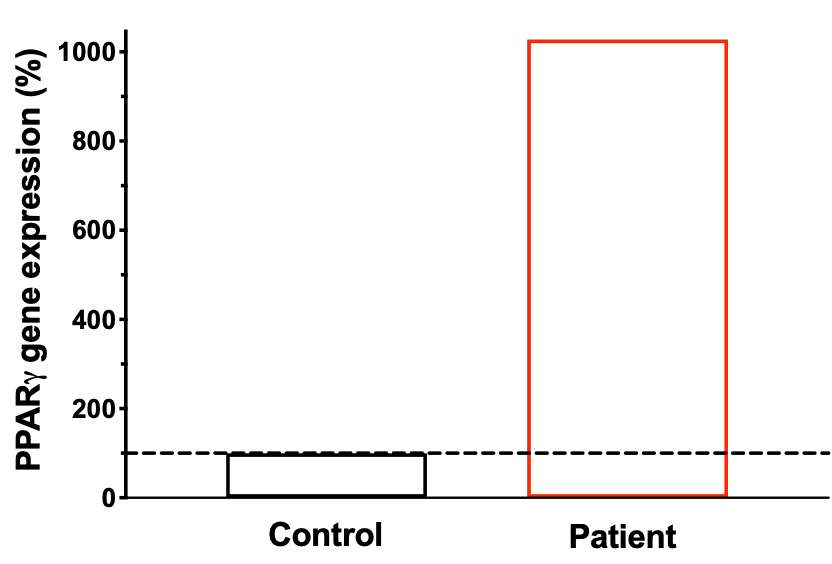


**Supplemental data 2**

**
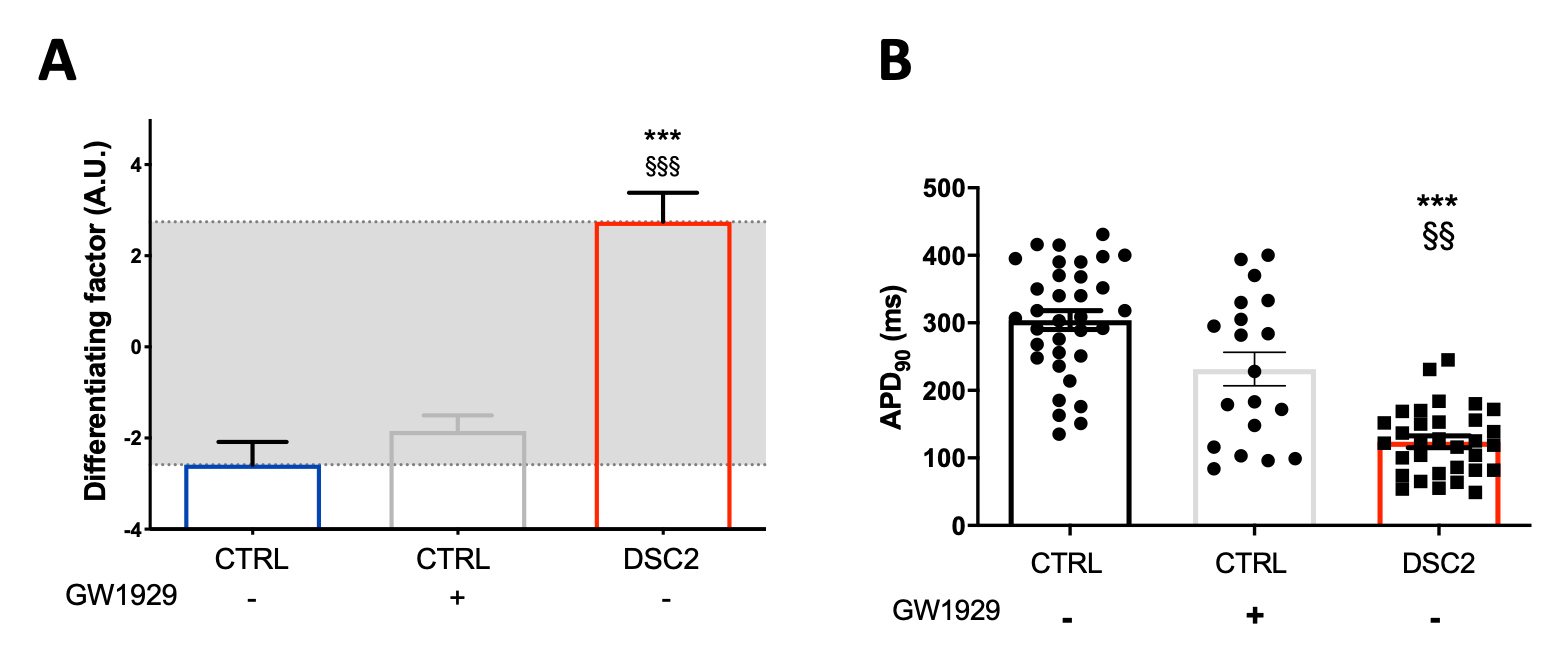
**

**Supplemental data 3**

**
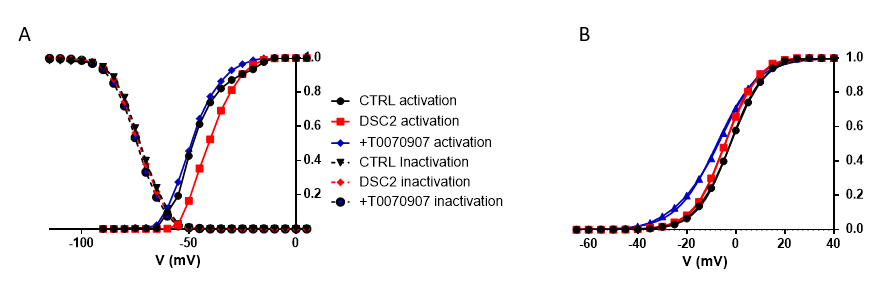
**

**Supplemental data 4**

**
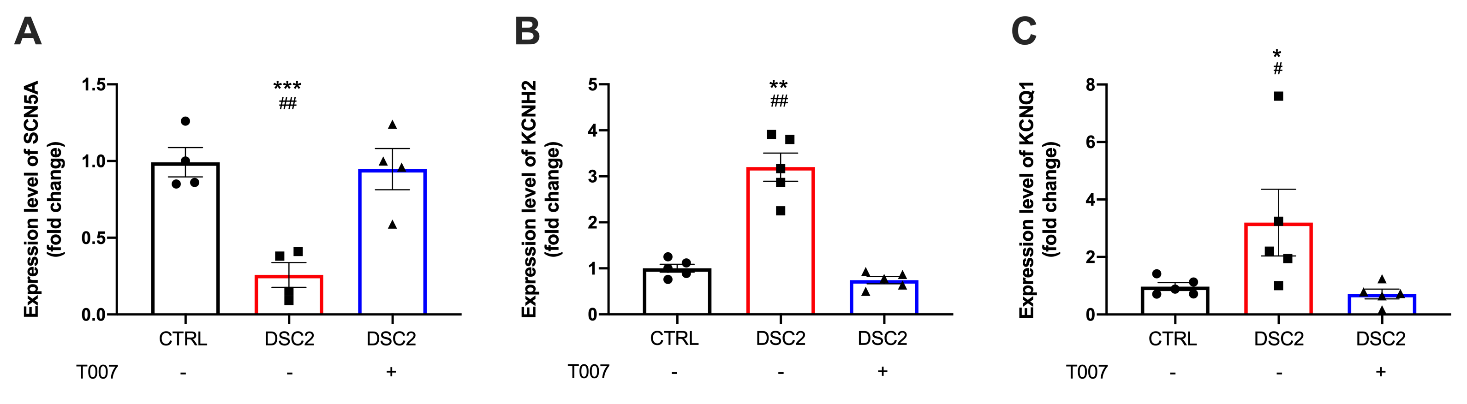
**

**Supplemental data 5**


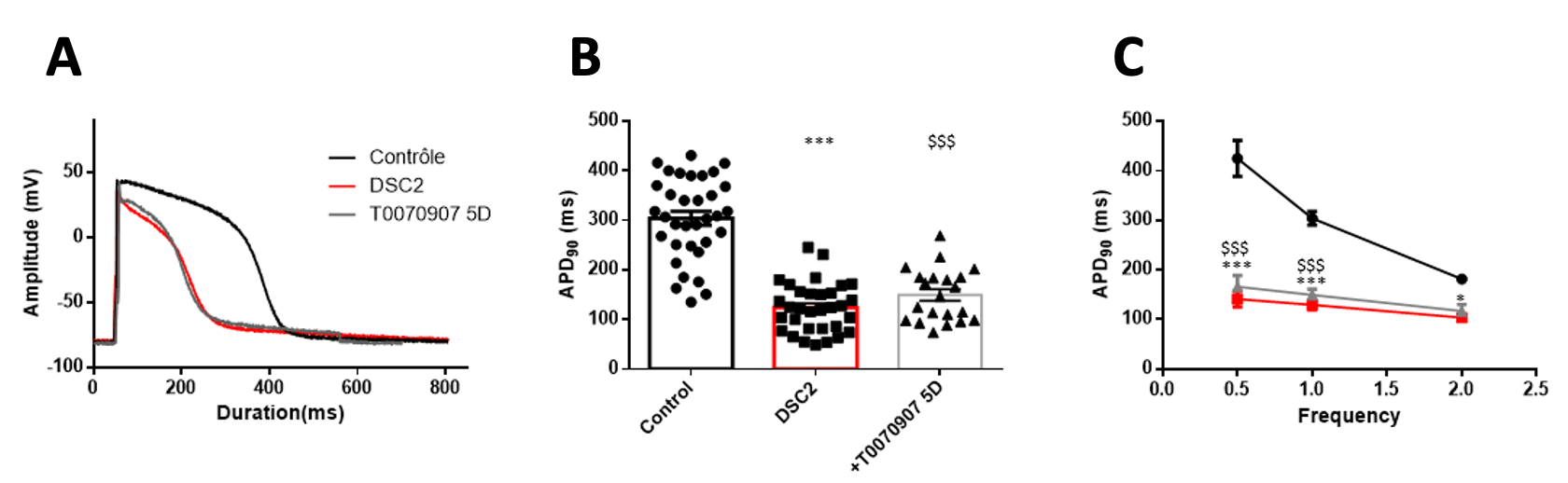


**Supplemental data 6**


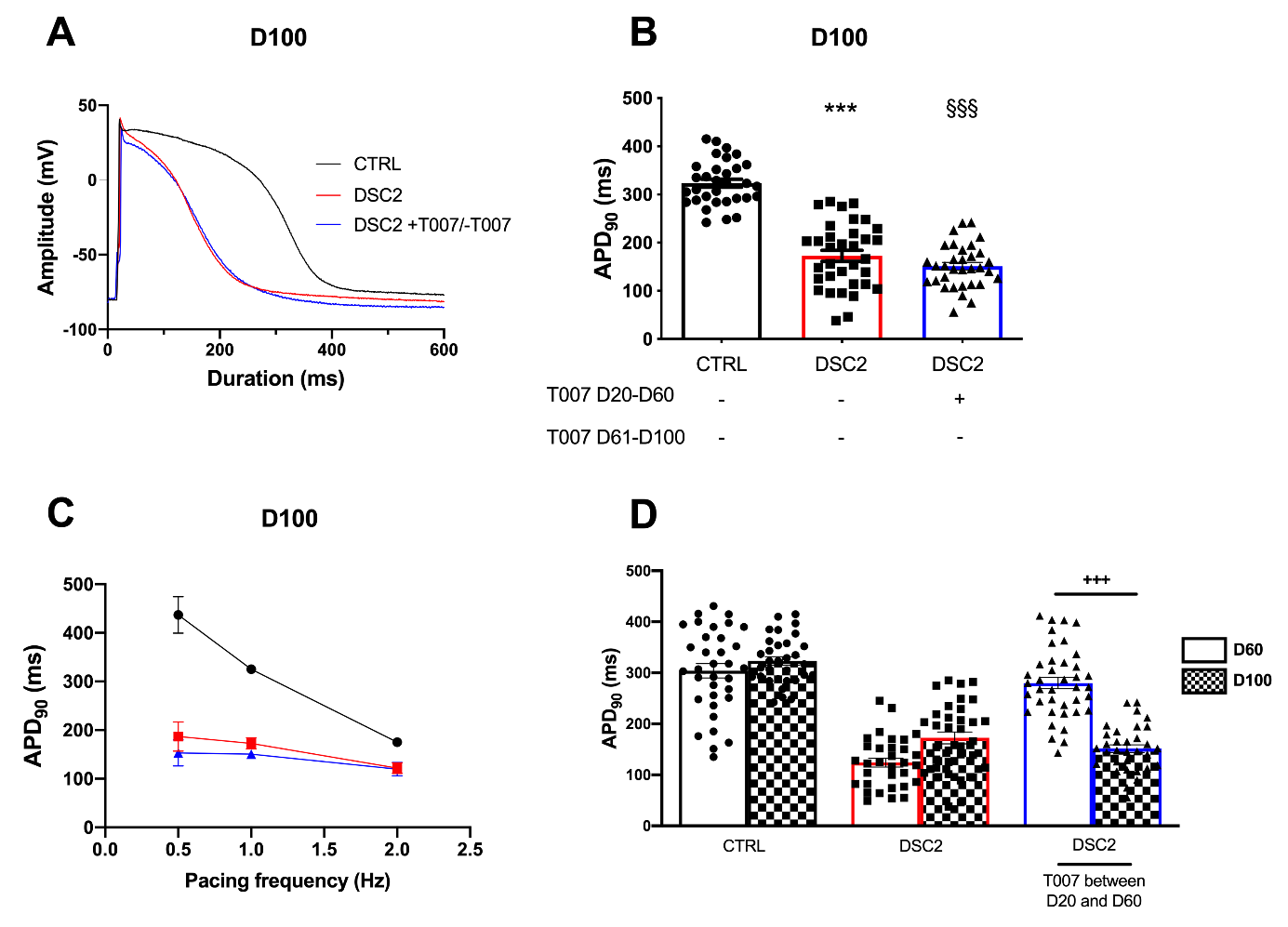


**Supplemental data 7**


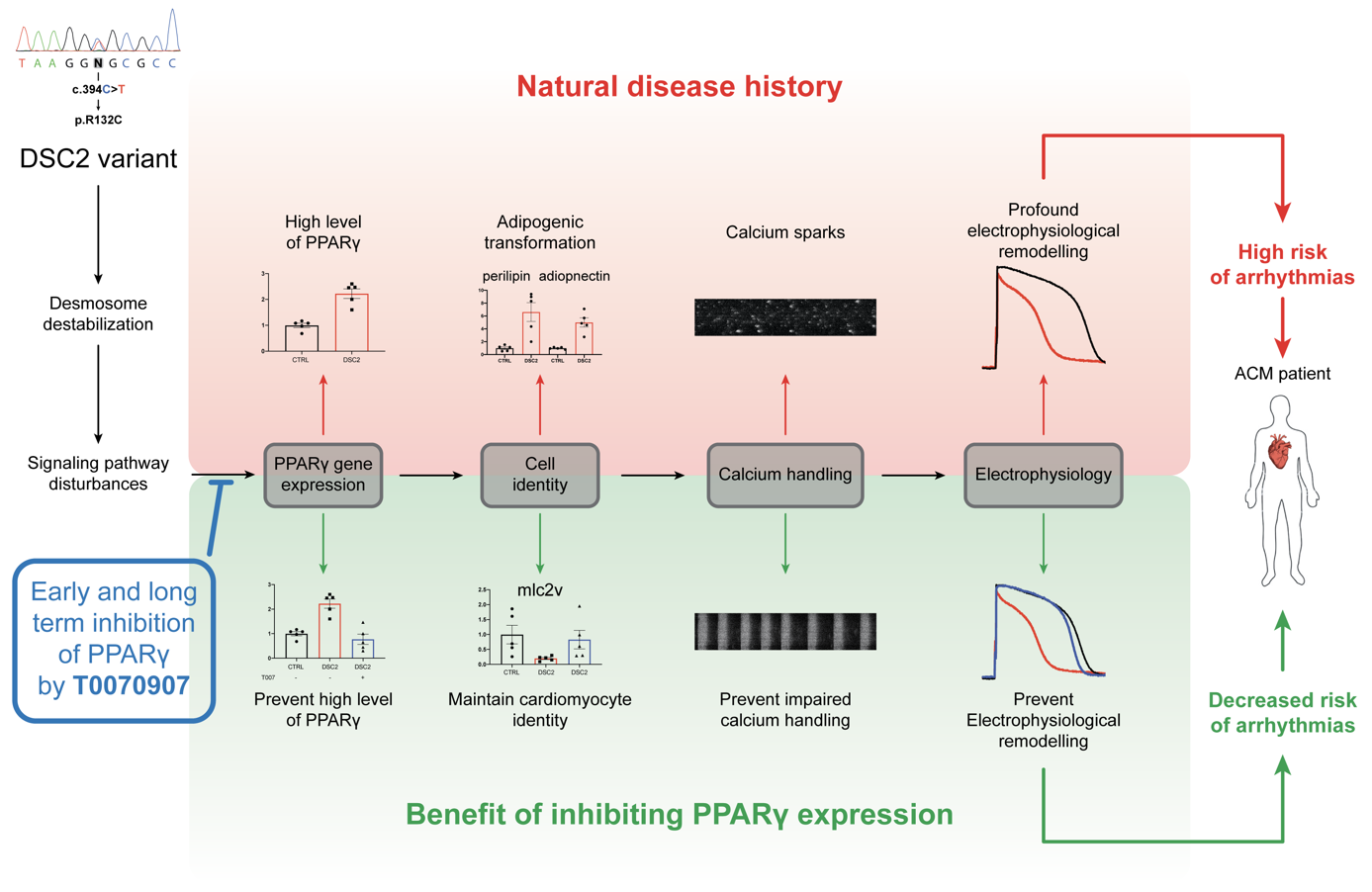

Supplement: Supplementary file 1 — Supporting Information Figure S1: PPARγ expression in patient DSC2 heart sample. Relative level of PPARγ in human heart sample from control and an arrhythmogenic cardiomyopathy (ACM) patient with a missense mutation (c.394C>T) in the DSC2 gene. Data obtained from transcriptome analysis (Affymetrix, HG‐U133_plus2 chips) Figure S2: Evaluation of PPARγ agonist in WT human‐induced pluripotent stem cells‐derived cardiomyocytes (hiPSC‐CM). (A) Differentiating factor of contractile property and (B) action potential (AP) duration at 90% of repolarisation (APD90) in WT hiPSC‐CM with or without 40 days of 5 μM GW1929. *Control versus DSC2, §DSC2 versus GW1929; §§ p < .01, ***,§§§ p < .001 (t‐test comparison) Figure S3: Evaluation of Nav biophysical parameters. (A) Voltage‐dependence of steady‐state activation and inactivation of Nav channels Figure S4: Evaluation of sodium and potassium channel expression. (A) The expression level of voltage‐gated Na+ channel alpha subunit (N = 4/group). Expression level study by reverse transcription‐quantitative polymerase chain reaction (RT‐qPCR) of potassium voltage‐gated channel subfamily H2 (B) and subfamily Q1 (C) (N = 4/group). Error bars represent the standard error of the mean (SEM). *Control versus DSC2, #DSC2 versus T007; *,# p < .05, **,## p< .01, *** p < .001 (Tuckey multiple comparisons test) Figure S5: Electrical activity of control and patient‐specific human‐induced pluripotent stem cells‐derived cardiomyocytes (hiPSC‐CMs) with or without PPARγ for 5 days. (A) raw trace illustrating the AP of control (black), patient‐specific hiPSC‐CM (red) and after incubation of T0070907 (grey), (B) Action potential (AP) duration at 90% of repolarisation (APD90), (C) adaptation of APD at 0.5, 1 and 2 Hz. Error bars represent the standard error of the mean (SEM). *Control versus DSC2, $control versus T007; * p < .05, ***,$$$ p < .001 (Tuckey multiple comparisons test) Figure S6: Inhibition of the PPARγ pathway is reversible. (A) Raw trace il [file CTM2-12-e748-s001.docx]
